# Spatio-temporal release of NGF and GDNF from multi-layered nanofibrous bicomponent electrospun scaffolds

**DOI:** 10.1007/s10856-018-6105-x

**Published:** 2018-06-28

**Authors:** Chaoyu Liu, Xiaohua Li, Feiyue Xu, Haibo Cong, Zongxian Li, Yuan Song, Min Wang

**Affiliations:** 1 Department of Mechanical Engineering, The University of Hong Kong, Pokfulam Road, Hong Kong, China; 2 Department of Research and Development, Shenzhen Gene Health Bio Tech Co., Ltd, Shenzhen, 518055 China; 3 Department of Reconstructive Microsurgery, Weihai Central Hospital, Weihai, 264400 China; 4 Department of Oncology, Weihai Central Hospital, Weihai, 264400 China

## Abstract

Scaffolds capable of providing dual neurotrophic factor (NTF) delivery with different release kinetics, spatial delivery of NTFs at different loci and topographical guidance are promising for enhanced peripheral nerve regeneration. In this study, we have designed and fabricated multi-layered aligned-fiber scaffolds through combining emulsion electrospinning, sequential electrospinning and high-speed electrospinning (HS-ES) to modulate the release behavior of glial cell line-derived growth factor(GDNF) and nerve growth factor (NGF). GDNF and NGF were incorporated into poly(lactic-co-glycolic acid) (PLGA) fibers and poly(D,L-lactic acid) (PDLLA) fibers, respectively. Aligned fibers were obtained in each layer of multi-layered scaffolds and relatively thick tri-layered and tetra-layered scaffolds with controlled layer thickness were obtained. Their morphology, structure, properties, and the in vitro release of growth factors were examined. Dual and spatio-temporal release of GDNF and NGF with different release kinetics from multi-layered scaffolds was successfully demonstrated. High separation efficiency by PDLLA fibrous barrier layer for spatial neurotrophic factor delivery from both tri-layered scaffolds and tetra-layered scaffolds was achieved.

## Introduction

Peripheral nerve tissue repair is still a challenge in reconstructive surgery although peripheral nerves are capable of regenerating to some extent. A nerve graft bridging the proximal and distal stumps is normally necessary to produce good regenerative outcomes in critical-size peripheral nerve injury [1]. Autografts with inherent supporting cells and intraluminal guidance exhibit superior capability of nerve regeneration and are seen as the gold standard for peripheral nerve tissue repair. However, the limitations of autografts, including insufficient sources, morbidity at donor site, mismatch in size, and necessity for multiple surgeries, have greatly hampered their clinical application [2].

In recent years, synthetic nerve guidance conduits (NGCs) mimicking the composition and structure of an autograft have been investigated as a promising alternative treatment for peripheral nerve injury. These artificial scaffold-based conduits may support axonal growth and provide various biological cues [3–6].

Neurotrophic factors (NTFs) including nerve growth factor (NGF) and glial cell line-derived neurotrophic factor (GDNF) are known to promote neuronal survival, axonal regeneration, and Schwann cells migration [7]. NGF and GDNF may be involved differently/ predominantly in physiologic processes following injury [8] and may promote axonal growth synergistically as well [9]. Contact guidance provided by topographical cues by NGCs can also promote neuronal growth and axonal extension [10, 11]. Therefore, scaffolds capable of achieving delivery of multiple NTFs with distinct release kinetics, spatial delivery of NTFs at different loci and topographical guidance are expected for much enhanced peripheral nerve regeneration.

The aim of this work was to investigate the feasibility delivery of GDNF and NGF with distinct release kinetics from multi-layered electrospun scaffolds. A combinatorial methodology including sequential electrospinning, high-speed electrospinning (HS-ES), dual-source dual-power electrospinning (DSDP-ES), and emulsion electrospinning was applied to construct fibrous scaffolds with desired structures and properties. Scaffolds with tri-layered and tetra-layered configurations were constructed in order to achieve dual and spatio-temporal delivery of GDNF and NGF. GDNF and NGF were incorporated into poly(lactic-co-glycolic acid) (PLGA) fibers and poly(d,l-lactic acid) (PDLLA) fibers, respectively, via emulsion electrospinning. Thickness of different layers in multi-layered scaffolds was controlled by the duration of fiber deposition. The morphology, structure and properties of scaffolds produced were investigated. The release profiles of both growth factors (GDNF and NGF) from tri-layered and tetra-layered scaffolds were established.

## Materials and methods

### Materials

PLGA (LA:GA=50:50) and PDLLA with molecular weight of 100 kDa (as indicated by their inherent viscosity 0.6–0.8 dL/g) were purchased from Lakeshore Biomaterials, USA. Chloroform was supplied by Uni Chem Co., Korea. The human β-NGF with Enzyme Linked Immunosorbent Assay (ELISA) Kit, human GDNF with ELISA Kit were purchased from Peprotech Inc. and R&D Systems, Inc., respectively. Span-80, phosphate buffered saline (PBS) tablets, heparin and bovine serum albumin (BSA) were Sigma-Aldrich products. Other chemicals were used as received.

### Fabrication of scaffolds

**Fig. 1:**
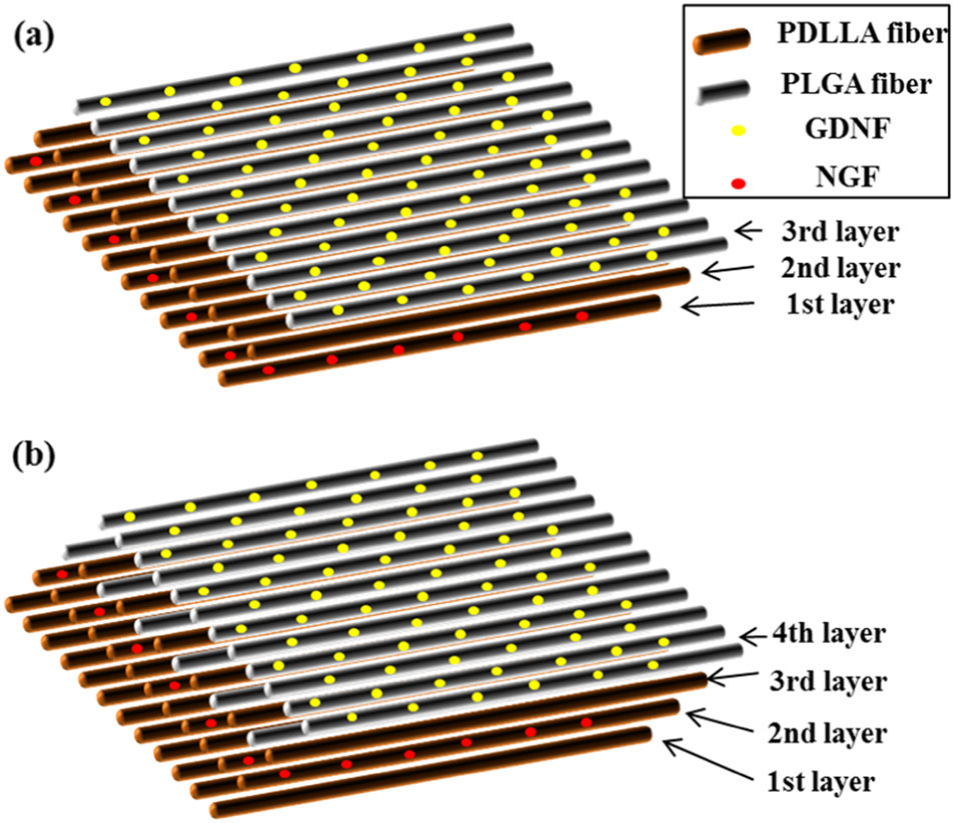
A sketch of multi-layered electrospun scaffolds: **a** tri-layered scaffolds, **b** tetra-layered scaffolds

Multi-layered scaffolds were produced through a combination of emulsion electrospinning, sequential electrospinning, HS-ES, and DSDP-ES. The formulations of emulsions for each layer in multi-layered scaffolds are listed in Table 1 and Table 2. For the preparation of water-in-oil (w/o) emulsions, PLGA or PDLLA dissolved in chloroform at a certain concentration was used as the oil phase and NGF or GDNF were dissolved in 0.5wt% BSA solution as the water phase. The volume ratio of the oil phase to the water phase was fixed at 10:1. 5wt% Span-80 (with respect to the weight of polymer used) was added in the polymer solution for the formation and stabilization of emulsions. The oil phase and water phase were mixed for 10 min through magnetic stirring at 300 rpm to form homogeneous w/o emulsions. The electrospinning parameters including applied voltage, inner diameter of needle tip, needle-to-collector distance and feeding rate of emulsions were optimized as 16 kV, 0.8 mm, 8 cm, and 2 mL/h, respectively. In the fabrication of tri-layered scaffolds, electrospun NGF/PDLLA fibers, PDLLA fibers, and GDNF/PLGA fibers were collected for 30 min to form the first layer, the middle layer, and the third layer, respectively. PDLLA fibers, NGF/PDLLA fibers, PDLLA and PLGA fibers, and GDNF/PLGA fibers constituted the first, second, third, and fourth layer of tetra-layered scaffolds, respectively. In order to control the thickness of each layer, the duration of electrospinning for each layer was strictly controlled, which was 30, 30, 15 and 30 min for the first layer to the fourth layer. The rotating direction of drum collector was regarded as aligned direction and the axial direction of drum collector as perpendicular direction. Schematic diagram for tri-layer and tetra-layer fibrous scaffolds is shown in Fig. 1.

**Table 1:** Composition of emulsions for producing tri-layered fibrous scaffolds

Tri-layered scaffold	1^st^ layer	2^nd^ layer	3^rd^ layer
Oil phase	1.5 g PDLLA 75 µL Span-80 10 mL CHCl_3_	2.5 g PDLLA 10 mL CHCl_3_	1.5 g PLGA 75 µL Span-80 10 mL CHCl_3_
Water phase	5 µg NGF 5 mg BSA 1 mL H_2_O		5 µg GDNF 5 mg BSA 1 mL H_2_O

**Table 2:** Composition of emulsions for producing tetra-layered fibrous scaffolds

Tetra-layered scaffold	1^st^ layer	2^nd^ layer	3^rd^ layer	4^th^ layer
Oil phase	2.5 g PDLLA 10 mL CHCl_3_	1.5 g PDLLA 75 µL Span-80 10 mL CHCl_3_	1.5 g PDLLA 75 µL Span-80 10 mL CHCl_3_; 1.5 g PLGA 75 µL Span-80 10 mL CHCl_3_	1.5 g PLGA 75 µL Span-80 10 mL CHCl_3_
Water phase		5 µg NGF 5 mg BSA 1 mL H_2_O	5 mg BSA 1 mLH_2_O; 5 mg BSA 1 mLH_2_O	5 µg GDNF 5 mg BSA 1 mLH_2_O

### Characterization of aligned-fiber scaffolds

Morphological and structural characterization of electrospun fibers and fabricated scaffolds was conducted. Freeze-dried samples of electrospun fibers and scaffolds were sputtered with a thin gold coating for 30s by a sputter coater (BEL-TACSCD005) and their morphology was examined using SEM (Hitachi S-4800 FEG SEM, Japan). SEM images were loaded into Image J (National Institute of Health, USA) and 100 fibers in each layer were randomly selected and their orientations were measured to determine the fiber alignment.

### Wettability of scaffolds

Wettability of electrospun fibrous scaffolds was investigated by measuring their water contact angles (WCAs) at room temperature with a WCA measuring machine (SL200B, Shanghai Solon Tech Inc Ltd, China). The WCA of each layer in both aligned and perpendicular directions were measured. Measurements were performed 3 s after a DI water drop was placed on the sample surface.

### Mechanical properties of multi-layered scaffolds

Tensile tests were performed using a bench top Instron mechanical testing machine (Model 5848, USA) with a load cell of 10 N and a cross-head speed of 2 mm/min. Each fibrous scaffold with similar thickness was cut into rectangular shape (5×30 mm) in the aligned direction for testing. The ultimate tensile strength (UTS), elastic modulus (EM), and elongation at break (EB) of scaffold samples were determined according to the recorded stress–strain curves. At least three replicates were tested for each type of fibrous scaffold and results were expressed as mean ± SD.

### In vitro release test of NGF and GDNF

Scaffold samples (about 10 mg each) were vertically inserted into specially designed container to form two separated chambers. Each chamber was filled with 3 mL release medium to fully immerse both sides of multi-layered scaffolds and incubated in shaking water bath at 37 °C. At pre-set times, 0.4 mL of supernatant was retrieved from each well and replaced by 0.4 mL of fresh release medium. The supernatant sample was frozen at −20 °C for further measurement or directly used for analysis. The concentration of growth factor in the supernatant was determined using ELISA kit. The release medium was prepared by adding 0.5% BSA, 0.05% Tween-20, 0.02% NaN_3_, and 0.1% heparin in PBS solution.

## Results

### Morphology and structure of scaffolds with aligned fibers

**Fig. 2:**
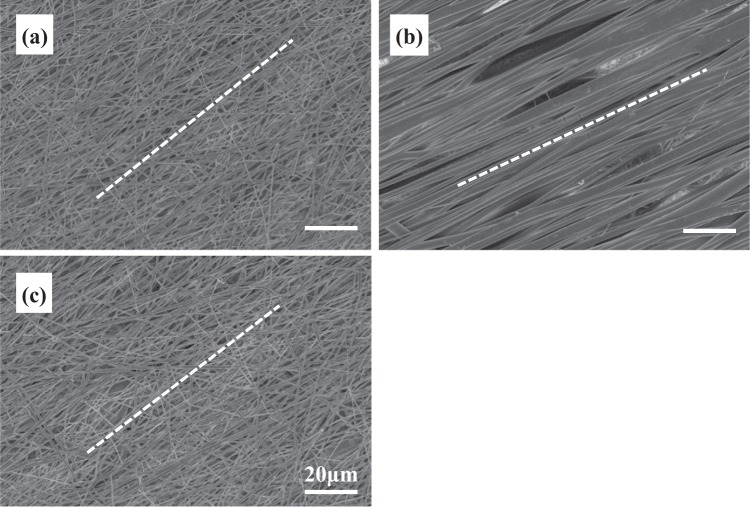
Morphology of tri-layered fibrous scaffold fabricated by DSDP-ES and sequential electrospinning under SEM at ×2500 magnification: **a** GDNF/PLGA top layer; **b** PDLLA middle layer; **c** NGF/PDLLA bottom layer

The morphology of different fibrous layers in tri-layered scaffolds was examined by SEM and is shown in Fig. 2. Robust fibers with smooth fiber surface were obtained in all layers. The average fiber diameter of GDNF/PLGA fibers in top layer, PDLLA fibers in middle layer and NGF/PDLLA fibers in bottom layer was 452 ± 98, 2928 ± 420, and 507 ± 102 nm, respectively. Porous and fibrous structure was observed in each layer in scaffolds. However, the apparent porosity in PDLLA middle layer was much smaller as compared to the other two layers (top and bottom) in tri-layered scaffolds.

**Fig. 3:**
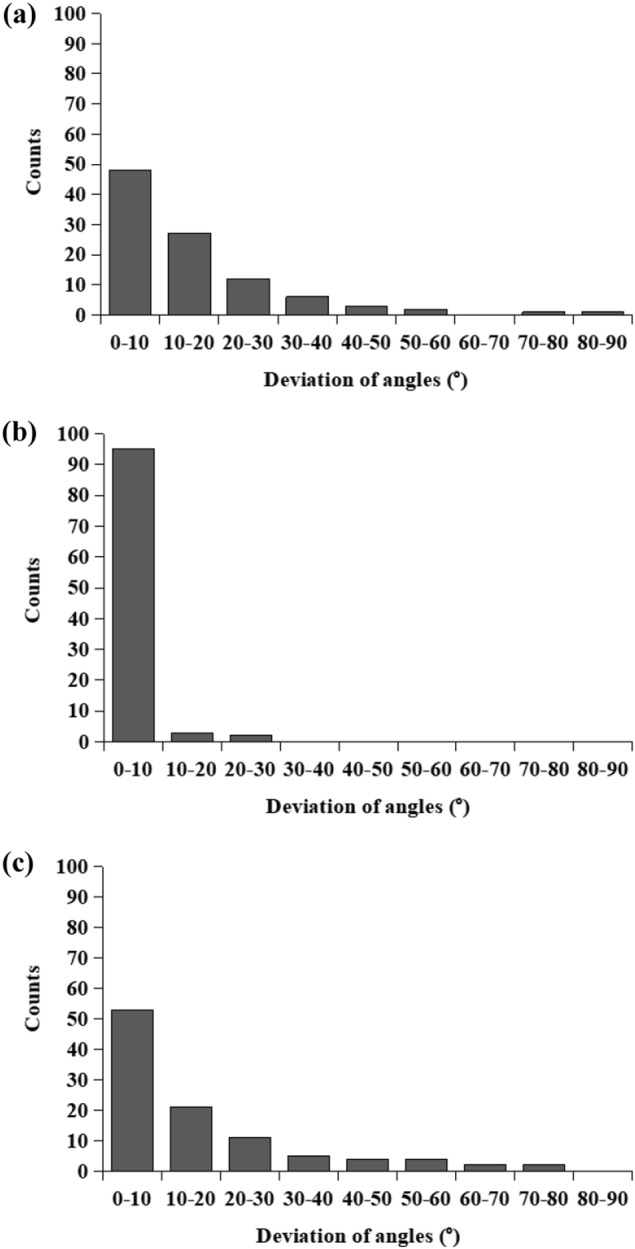
Distribution of fiber alignment in different layers of tri-layered scaffolds: **a** GDNF/PLGA top layer, **b** PDLLA middle layer, **c** NGF/PDLLA bottom layer

Fiber alignment could be clearly noticed in each layer of the scaffold which was deposited on high-speed rotating drum collector and the average fiber orientation is delineated as dotted line in Fig. 2. The fiber alignment was quantified by calculating frequencies of fiber orientations and is shown in Fig. 3. A narrower range of fiber orientation (deviation of angles) represented a higher fiber alignment. The highest fiber alignment was observed in PDLLA middle layer with more than 90% of fibers oriented within 10° deviating from aligned direction. The lowest fiber alignment was observed in GDNF/PLGA top layer with around 30% of fibers oriented within 10° deviating from aligned direction.

**Fig. 4:**
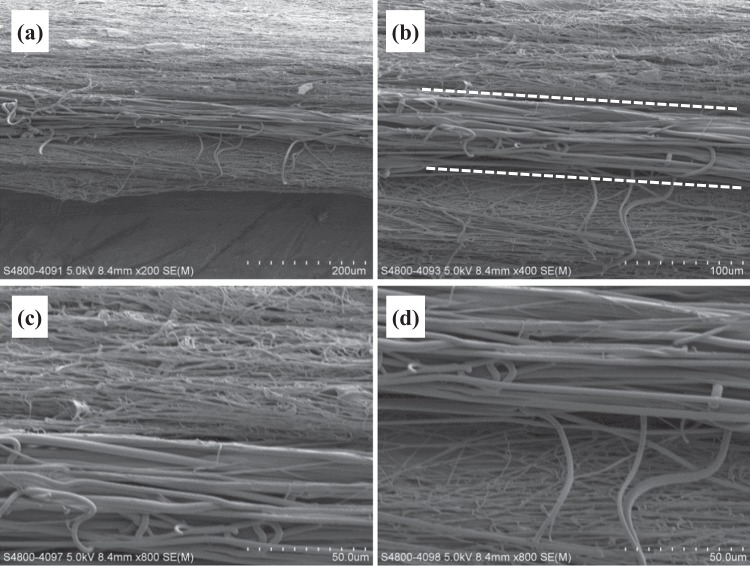
Cross-sectional view of tri-layered fibrous scaffolds under SEM: **a** magnification: 200, **b** magnification: 400, **c** layer boundary between top layer and middle layer (magnification: 800), **d** layer boundary between middle layer and bottom layer (magnification: 800)

The fiber alignment in NGF/PDLLA bottom layer was higher as compared to that in top layer (GDNF/PLGA layer) but lower than that in middle layer (PDLLA layer). Through sequential electrospinning and emulsion electrospinning, tri-layered scaffolds composed of GDNF/PLGA top layer, PDLLA middle layer, and NGF/PDLLA bottom layer were obtained. The cross-sectional morphology of tri-layered scaffolds and boundaries between different layers were examined by SEM and are shown in Fig. 4. The layer boundaries between different layers are marked with dotted lines. The fiber alignment in different layers was more evident from the cross-section view. The average thickness of tri-layered scaffolds was 262 ± 16 μm and the average thickness of top layer, middle layer, and bottom layer was 78 ± 3 μm, 86 ± 13 μm, and 82 ± 4 μm, respectively.

**Fig. 5:**
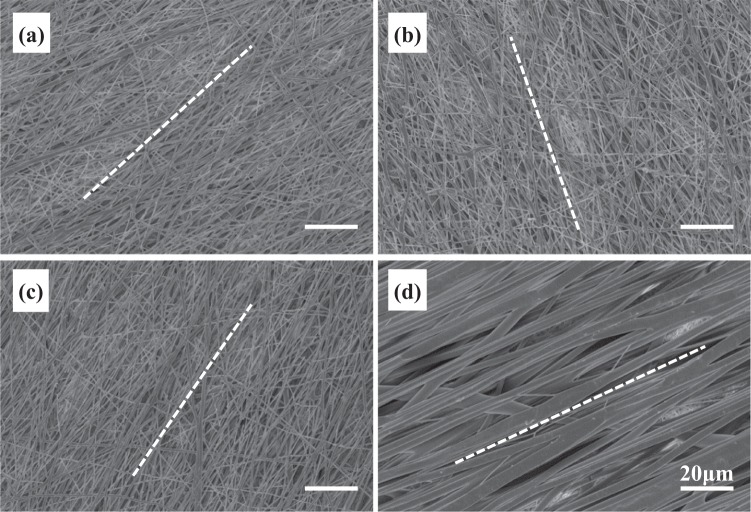
Morphology of tetra-layered fibrous scaffold fabricated by sequential electrospinning and DSDP-ES under SEM at ×2500 magnification: **a** GDNF/PLGA top layer; **b** mixed layer of PLGA fibers and PDLLA fibers; **c** NGF/PDLLA third layer; **d** PDLLA bottom layer

The morphology of different layers in tetra-layered scaffolds was examined by SEM and is shown in Fig. 5. Robust fibers with smooth fiber surface were also obtained in all layers. The average fiber diameter of GDNF/PLGA fibers in top layer, mixed layer of PLGA fibers and PDLLA fibers (the second layer), NGF/PDLLA fibers in third layer, and PDLLA fibers in bottom layer was 467 ± 108, 486 ± 117, 498 ± 96, and 2860 ± 494 nm, respectively. Porous and fibrous structure was observed in each layer. The apparent porosity in PDLLA bottom layer was much smaller as compared to the other three layers in tetra-layered scaffolds.

**Fig. 6:**
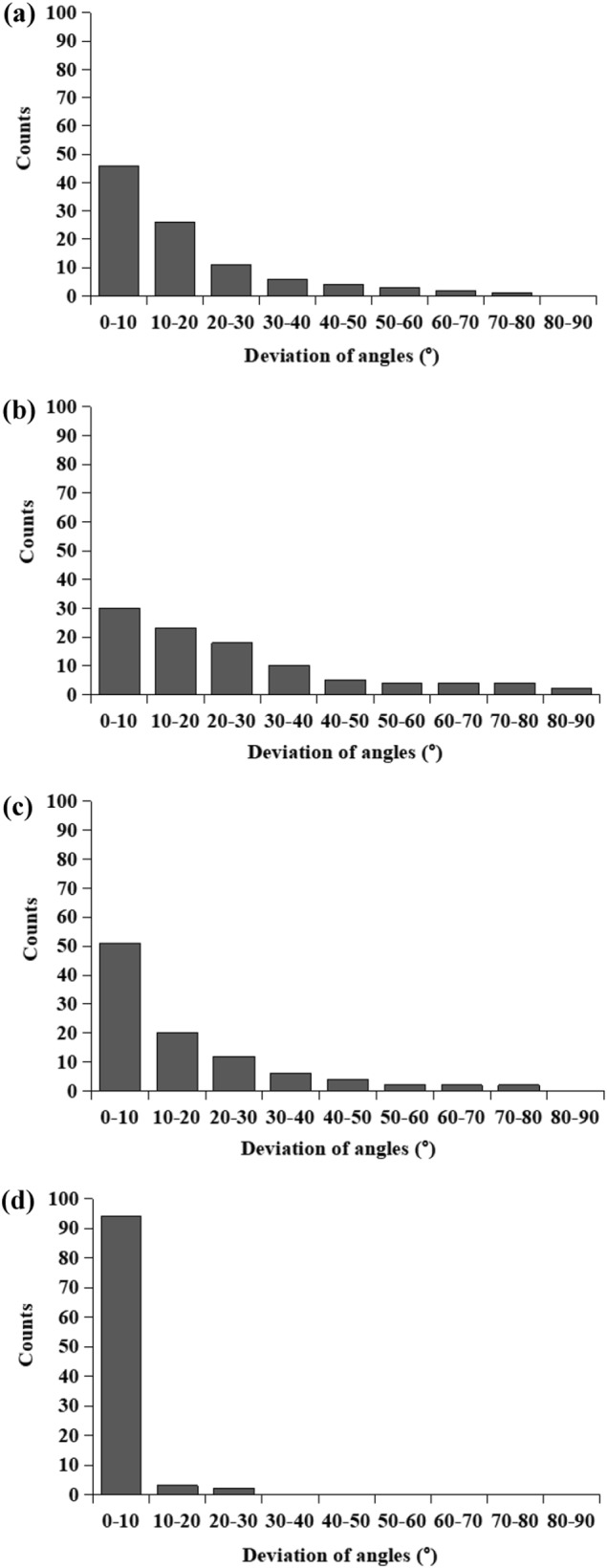
Distribution of fiber alignment in different layers of tetra-layered scaffolds: **a** GDNF/PLGA top layer, **b** mixed layer of PLGA fibers and PDLLA fibers, **c** NGF/PDLLA third layer, and **d** PDLLA bottom layer

Fiber alignment could be clearly noticed in each layer deposited on high-speed rotating drum collector and the average fiber orientation is delineated as dotted line in Fig. 5. The fiber alignment in each fibrous layer was quantified and is shown in Fig. 6. The highest fiber alignment was observed in PDLLA middle layer with more than 90% of fibers oriented within 10° deviating from aligned direction. The lowest fiber alignment was observed in GDNF/PLGA top layer with around 30% of fibers oriented within 10° deviating from aligned direction. The fiber alignment in mixed layer of PLGA fibers and PDLLA fibers was higher as compared to that in GDNF/PLGA top layer but lower as compared with that in NGF/PDLLA third layer.

**Fig. 7:**
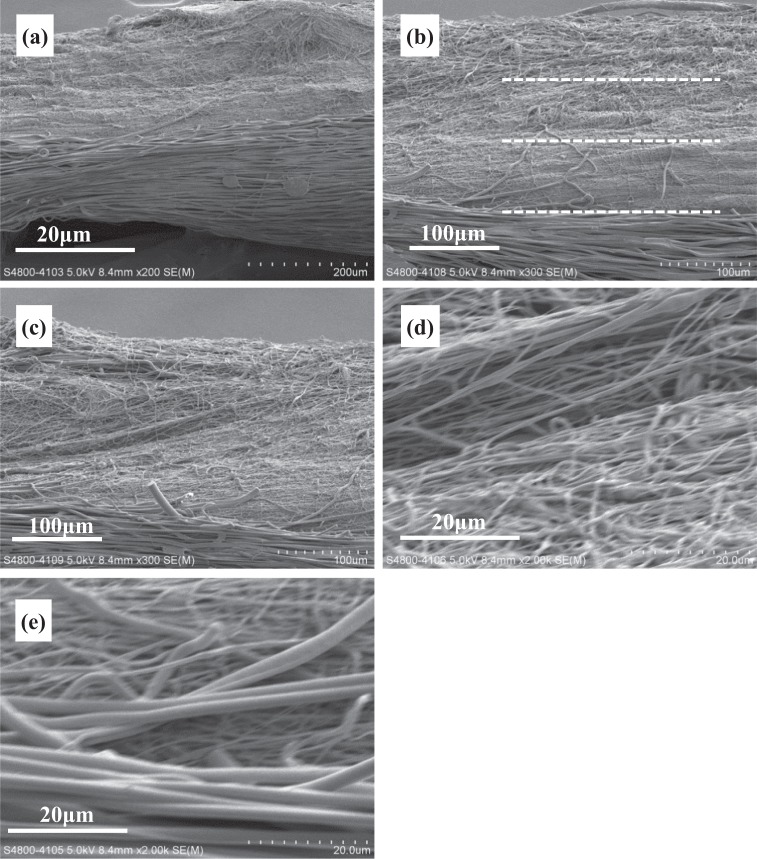
Cross-sectional view of tetra-layered fibrous scaffolds under SEM: **a** magnification: 200; **b**, **c** magnification: 300; **d** layer boundary between top layer and second layer at magnification: 2000; **e** layer boundary between third layer and bottom layer at magnification: 2000

Through sequential electrospinning and emulsion electrospinning, tetra-layered scaffolds composed of GDNF/PLGA top layer, mixed layer of PGLA fibers and PDLLA fibers, NGF/PDLLA third layer, and PDLLA bottom layer were also obtained. The cross-sectional morphology of the tetra-layered scaffolds and boundaries between different layers were examined by SEM and are shown in Fig. 7. The layer boundaries between different layers are marked with dotted lines (Fig. 7b). The average thickness of tetra-layered scaffolds was 310 ± 16 μm and the average thickness of top layer, second layer, third layer, and bottom layer was 72 ± 3 μm, 65 ± 3 μm, 81 ± 6 μm, and 82 ± 4 μm, respectively.

### Wettability of fibrous scaffolds

The wettability of both tri-layered and tetra-layered fibrous scaffolds was measured by water contact angle (WCA) tests. The WCA of each layer in both directions was measured.

**Fig. 8:**
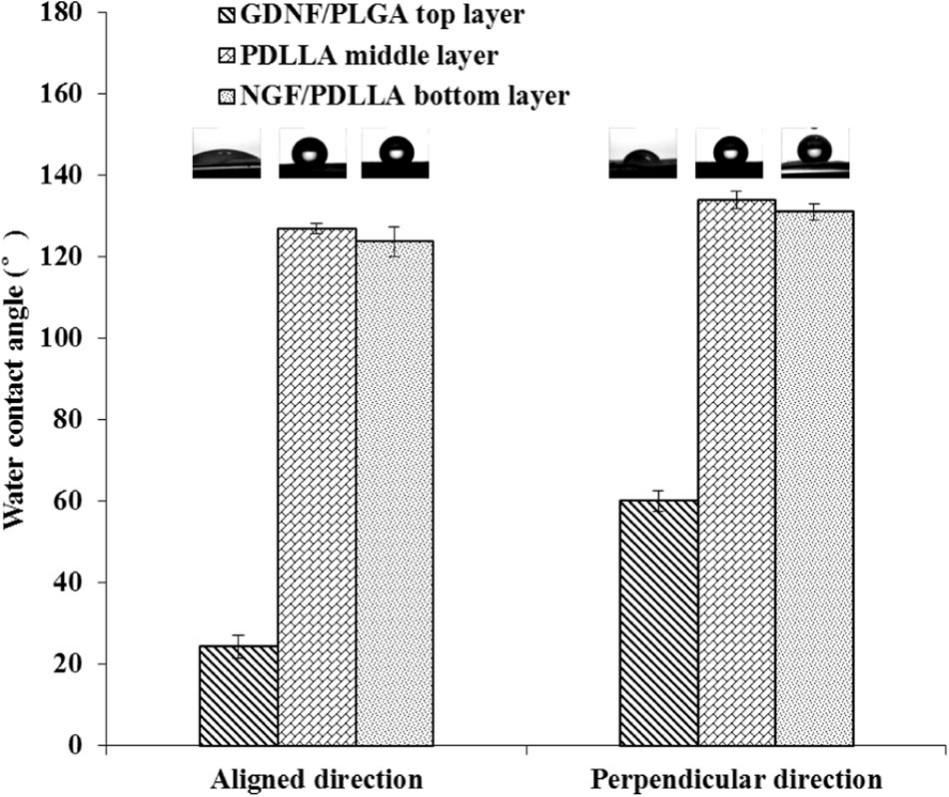
Water contact angles of different layers in different directions in tri-layered fibrous scaffolds

As shown in Fig. 8, in tri-layered fibrous scaffolds, the average WCA of GDNF/PLGA top layer, PDLLA middle layer and NGF/PDLLA bottom layer in aligned direction was 24.4°, 126.9°, and 123.7°, respectively. The average WCA of different layers from top layer to bottom layer in perpendicular direction was 60.1°, 133.9°, and 131.0°, respectively. The morphology of water droplets on each scaffold sample captured by camera is also displayed in Fig. 8. The highest WCA was observed in the PDLLA middle layer and much lower WCA was noticed in the GDNF/PLGA top layer in both directions. And the WCA of each layer in aligned direction was evidently lower as compared to the WCA of their counterparts in perpendicular direction.

**Fig. 9:**
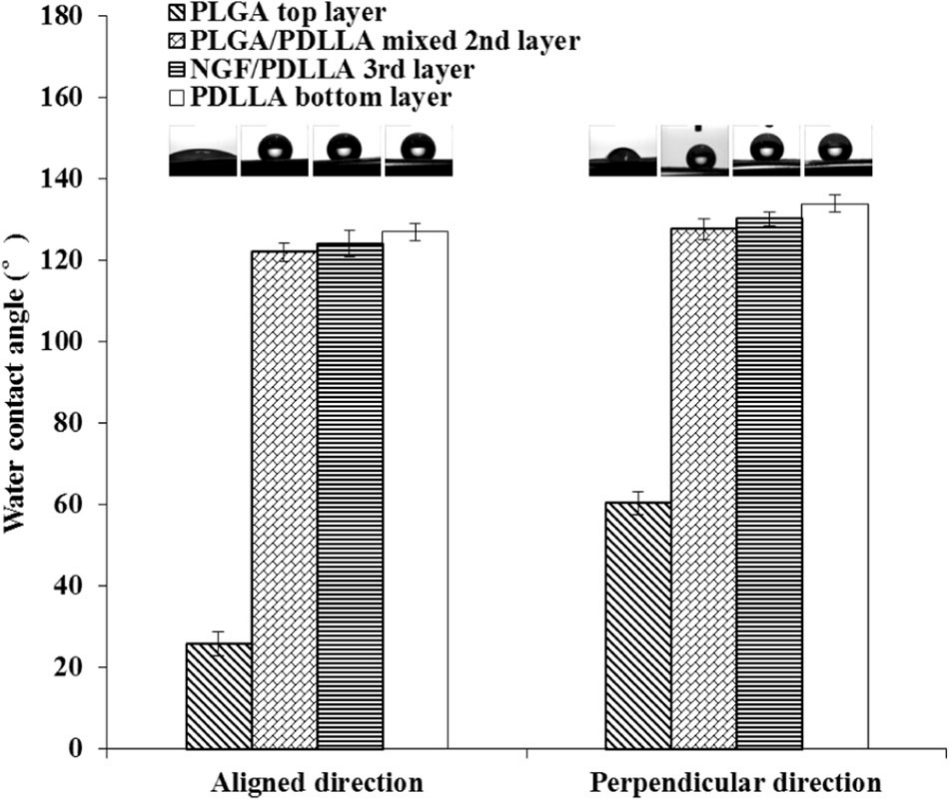
Water contact angles of different layers in different directions in tetra-layered fibrous scaffolds

As shown in Fig. 9, in tetra-layered fibrous scaffolds, the average WCA of GDNF/PLGA top layer, mixed layer of PLGA fibers and PDLLA fibers, NGF/PDLLA third layer, and PDLLA bottom layer in aligned direction was 25.8°, 122.0°, 124.0°, and 126.9°, respectively. The average WCA of different layers from top layer to bottom layer in perpendicular direction was 60.3°, 127.6°, 130.1°, and 133.9°, respectively. The morphology of water droplets on each scaffold sample captured by camera is also displayed in Fig. 9. Much lower WCA was observed in GDNF/PLGA top layer in both directions as compared to the WCA of other layers. The WCA of each layer increased from the top layer to the bottom layer and the highest WCA was observed in the PDLLA bottom layer in both directions. And the WCA of each layer in aligned direction was evidently lower as compared to the WCA of their counterparts in perpendicular direction.

### Mechanical properties of fibrous scaffolds

**Fig. 10:**
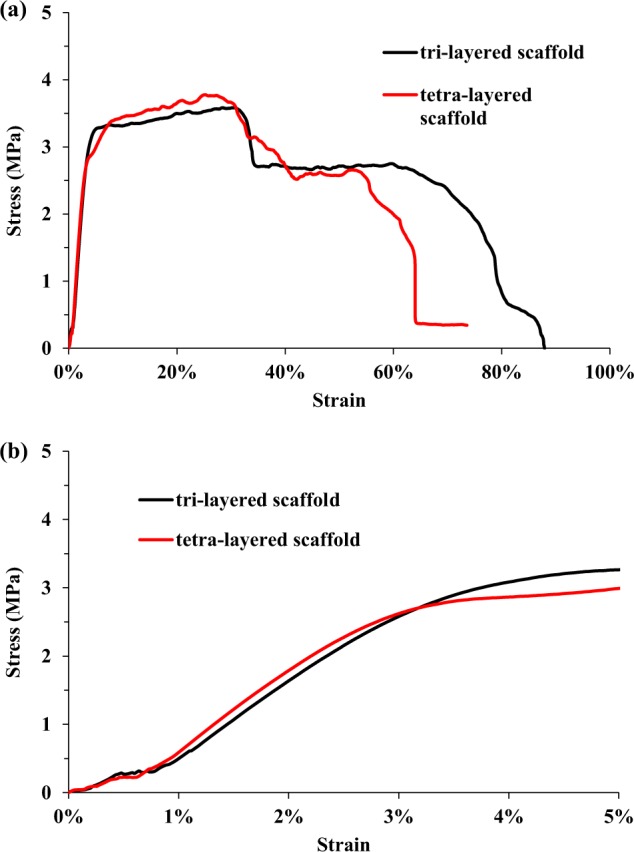
Stress–strain curves of tri-layered scaffolds and tetra-layered scaffolds obtained by tensile tests in aligned direction: **a** full stress–strain curves, **b** initial portions of stress–strain curves

Tensile tests of tri-layered and tetra-layered fibrous scaffolds in aligned direction were performed and typical stress–strain curves are shown in Fig. 10. Initial portions of stress–strain curves including elastic region within 5% strain are also displayed. The mechanical properties of tri-layered and tetra-layered fibrous scaffolds including UTS, EM, and EB were measured using the stress–strain curves. The EM, UTS, and EB of tri-layered fibrous scaffolds were 101.2 ± 4.8 MPa, 3.6 ± 0.3 MPa, and 88 ± 7%, respectively. The EM, UTS, and EB of tetra-layered fibrous scaffolds were 97.6 ± 4.9 MPa, 3.8 ± 0.3 MPa, and 74 ± 6%, respectively.

### In vitro release profiles of growth factors

**Fig. 11:**
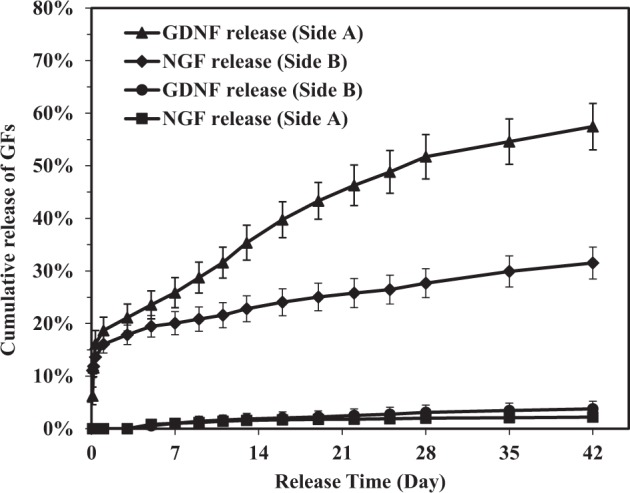
Cumulative release of NGF and GDNF from different sides of tri-layered fibrous scaffolds in 42-day in vitro release experiments

**Fig. 12:**
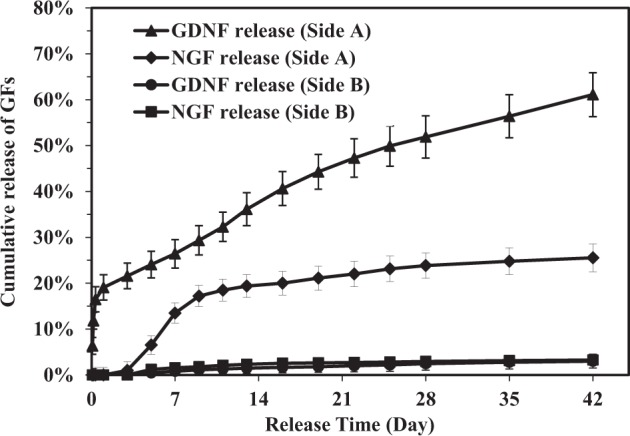
Cumulative release of NGF and GDNF from different sides of tetra-layered fibrous scaffolds in 42-day in vitro release experiments

The in vitro release experiments for growth factors from tri-layered and tetra-layered scaffolds were conducted and the release profiles of GDNF and NGF are shown in Figs. 11 and 12.

In tri-layered scaffolds, the GDNF/PLGA top layer side is designated as side A and the NGF/PDLLA bottom layer side is designated as side B. In the experiments, about 16.1% of NGF was released within the first 24 h, followed by a much slower and sustained release from side B. After 42 days, the cumulative release percentage reached 31.5%. However, only 2.2% of NGF was released from side A at the end of this release period. A much faster release behavior was observed for GDNF. The cumulative release percentage of GDNF from side A reached 18.7% within 1 day, which was slightly higher than that of NGF. Afterwards, the cumulative release ascended steadily, reaching 57.5% at the end of 42 days. And 3.8% of GDNF was released from side B at the end of the 42-day release period. An increase in GNDF release rate was noticed at around 14 days of the in vitro release experiments. The in vitro release results revealed that sustained release of GDNF and NGF from tri-layered fibrous scaffolds was obtained and the spatial release and delivery of GDNF and NGF at different sides of tri-layered scaffolds was successfully achieved.

In tetra-layered scaffolds, the GDNF/PLGA top layer side is designated as side A and PDLLA bottom layer side is designated as side B. The NGF release within the first 24 h from side A could be hardly detected. An increase in NGF release rate was noticed from day 3 to day 7. The cumulative release percentage of NGF reached 13.5% after 7 days, followed by a much slower and sustained release. After 42 days, the cumulative release percentage went up to 25.6%. However, only 3.3% of NGF was released from side B at the end of the 42-day release period. A much faster release behavior was observed for GDNF. The cumulative release percentage of GDNF from side A was 19.1% after 24 h. Afterwards, the cumulative release ascended steadily, reaching 61.1% at the end of 42 days. Only 3.0% of GDNF was released from side B at the end of 42-day release period. An increase in GNDF release rate was noticed at around 14 days of the in vitro release experiments. The in vitro release results revealed that dual and sustained release of GDNF and NGF from side A of tetra-layered fibrous scaffolds was obtained. A sequential release of GDNF and NGF was also achieved as GDNF was released first followed by NGF release. The release and delivery of GDNF and NGF was mainly confined at side A of tetra-layered scaffolds as growth factors were released at very low level at side B.

## Discussion

Peripheral nerve injury is one of the major challenges in reconstructive surgery. Although peripheral nerves are capable of regenerating themselves to some extent, when there is a severe peripheral nerve defect, a nerve graft is necessary [12, 13]. Under normal conditions, surgeons need to suture from end to end and bridge the proximal and distal stumps to produce good regenerative outcomes. Nerve segments from another body part of the patient are harvested to bridge the nerve gap. Currently, this is the gold standard of procedure for peripheral nerve tissue repair. However, the limited availability of autologous nerve segments, the potential morbidity at donor site, the mismatch in size and the necessity for multiple surgeries, have greatly hindered their clinical application [14].

In natural peripheral nerve tissues, bundles of axons align along the nerve channel, which are surrounded and supported by other cells including Schwann cells and glial cells. Neurotrophic factors such as GDNF and NGF secreted from surrounding cells can promote neuronal growth and axonal extension [8]. The development of synthetic artificial NGCs mimicking the composition, structures, and functions of an autograft to provide enhanced peripheral nerve regeneration is urgent. In peripheral nerve repair, different amounts of biochemical cues including NGF and GDNF are required to provide therapeutic effect [7]. Low amounts of NGF (<1 ng) is advantageous as high amounts of NGF can result in extensive branching of regenerated axons leading to aberrant target innervation; however, high amounts of GDNF (1–10 ng) can induce optimal axonal growth. It was also found that co-delivery of GDNF and NGF promoted both axonal elongation and branching, which was better than using GDNF or NGF alone [9]. In addition to biochemical cues, topographical cues can also enhance neurite outgrowth, axonal regeneration and promote peripheral nerve tissue repair [15, 16]. Therefore, biocompatible and biodegradable scaffold-based NGCs providing dual delivery and spatio-temporal delivery of GDNF and NGF with distinct release kinetics in a controlled manner, as well as topographical contact guidance are of interest and importance for axonal regeneration and peripheral nerve tissue repair.

Electrospun fibrous scaffolds have been increasingly employed for various tissue-engineering applications owing their unique properties. Electrospinning process provides diversity for fabricating tissue-engineering scaffolds with micro to nanoscale architectures, high porosity resembling natural extracellular matrix and high surface area to volume ratio. Electrospun fibrous scaffolds can induce excellent cell responses including cell adhesion, migration, and proliferation. Abundant materials can be electrospun including both natural and synthetic polymers such as gelatin, chitosan, silk fibroin, poly (ε-caprolactone) (PCL), PLGA, and PDLLA [17–22]. Electrospun fibers with various morphology and structures can be produced by different electrospinning techniques and utilized as delivery vehicles for various functional biomolecules including drugs, proteins, and even DNA and RNA [23–26]. Our previous study shows that emulsion electrospinning is an efficient method to incorporate growth factors with high encapsulation efficiency, and preserve their bioactivity [27].

In this study, multi-layered electrospun scaffolds composed of aligned fibers providing dual and spatio-temporal delivery of GDNF and NGF with distinct release kinetics were successfully produced through a combinatorial methodology including emulsion electrospinning, sequential electrospinning, HS-ES, and DSDP-ES. Biocompatible and biodegradable materials—PLGA and PDLLA—with different degradation rates were used as matrices of fibers in scaffolds. Multi-layered structures of scaffolds were realized by sequential deposition of electrospun fibers on a rotating collector. Aligned fibers in scaffolds were obtained by HS-ES. GDNF and NGF were incorporated into PLGA fibers and NGF fibers, respectively, through emulsion electrospinning. GDNF/PLGA fibers and NGF/PDLLA fibers were used for different layers, forming multi-layered structures in both tri-layered and tetra-layered scaffolds.

Fibrous scaffolds with aligned-fiber topography are sought in nerve tissue repair as aligned fibers can support cell proliferation, guide cell alignment, promote neurite outgrowth, and even induce neural differentiation [10, 28–30]. However, due to the bending instability of electrospun fibers during electrospinning, scaffolds with non-woven structures composed of randomly arranged fibers are normally obtained through conventional electrospinning. Other electrospinning techniques including but not limited to electrospinning with auxiliary electrodes, and magnetic field-assisted electrospinning (MFAES) have also been reported to fabricate aligned electrospun fibers by surmounting the bending instability of electrospun fibers [29, 31–33]. In this study, the mechanical tractive force provided by HS-ES overcame the other forces including electrostatic force, surface tension, and resistance force from shearing, alleviated, or eliminated bending instability and consequently facilitated the alignment of electrospun fibers.

The average fiber diameters of PDLLA fibers, which were 2928 ± 420 nm and 2860 ± 494 nm in tri-layered and tetra-layered scaffolds, respectively, were much larger than that of other types of fibers which were in the range of 450–10 nm. The PDLLA layer with much thicker fiber diameter was designed intentionally as a barrier layer. Aligned fibers were obtained in both tri-layered and tetra-layered scaffolds (Figs. 2 and 5). However, the fiber alignment in different layers was different (Figs. 3 and 6). In tri-layered scaffolds, the fiber alignment in PDLLA middle layer apparently outperformed that in GDNF/PLGA top layer and NGF/PDLLA bottom layer. A small decrease in fiber alignment was noticed in the top layer as compared to the bottom layer. In tetra-layered scaffolds, a similar situation was found. The fiber alignment in PDLLA bottom layer was similar to that in PDLLA middle layer in tri-layered scaffolds and exceeded that in other layers of tetra-layered scaffolds. A small decrease in fiber alignment was also noticed in GDNF/PLGA top layer as compared to NGF/PDLLA third layer. The lowest fiber alignment was observed in second layer composed of PLGA fibers and PDLLA fibers which was produced by DSDP-ES. It may be concluded that fiber alignment was disturbed with the increase of scaffold thickness to limited extent. DSDP-ES also perturbed the alignment of electrospun fibers to a certain degree. Under the investigated conditions of HS-ES, thick fiber diameter helped improve the alignment of electrospun fibers. It was believed that the decrease in fiber alignment with the increase of scaffold thickness was due to repulsive residual charges and the insulating effects of previously deposited electrospun fibers [34, 35]. It was speculated that mutual electrostatic repulsion between PLGA and PDLLA fibers during DSDP-ES also disturbed the fiber alignment. On the contrary, large inertia of thick fibers reducing bending instability might facilitate the alignment of electrospun fibers.

Relatively thick tri-layered and tetra-layered scaffolds of large size were obtained with an average thickness of 262 ± 16 μm and 310 ± 16 μm, respectively. The deposition time for each layer was maintained at 30 min except that for mixed layer composed of PLGA fibers and PDLLA fibers through DSDP-ES in tetra-layered scaffolds which was 15 min. The average thickness of top layer, middle layer and bottom layer in tri-layered scaffolds was similar, which was 78 ± 3 μm, 86 ± 13 μm, and 82 ± 4 μm, respectively. The average thickness of top layer, second layer, third layer, and bottom layer in tetra-layered scaffolds was 72 ± 3 μm, 65 ± 3 μm, 81 ± 6 μm, and 82 ± 4 μm, respectively. In tetra-layered scaffolds, a small decrease of layer thickness was noticed in the second layer and a slight decrease of layer thickness was observed in the top layer as compared to the layer thickness of the third and bottom layers. Although there was a concern about decrease of collection rate with thicker scaffold thickness due to repulsive residual charges and the insulating effects of previously deposited fibers, the results in this investigation revealed that the collection rate of fibers was influenced by scaffold thickness only to a very limited extent. An average fiber collection rate of approximately 160 μm/h (thickness over time) was achieved. These results demonstrated the feasibility of producing multi-layered scaffolds with controlled layer thickness through a combination of sequential electrospinning, HS-ES and DSDP-ES.

Wettability is an important characteristic of scaffolds, which can influence the cell responses such as cell adhesion and cell spreading [36]. There is no consensus on optimal wettability of scaffolds for all types of cells. The wettability of each layer in tri-layered scaffolds varied from each other in both directions. The GDNF/PLGA top layer was much more hydrophilic as compared to PDLLA middle layer and NGF/PDLLA bottom layer, which was mainly attributed to the hydrophilicity/hydrophobicity of polymers as PLGA is less hydrophobic than PDLLA. It appeared that thicker fiber diameter in PDLLA middle layer resulted in higher WCA as compared to that in NGF/PDLLA bottom layer. Similarly, this was observed in tetra-layered scaffolds when comparing the WCAs of NGF/PDLLA third layer and PDLLA bottom layer. A gradient wettability was observed in tetra-layered scaffolds in both directions as WCA increased from top layer to bottom layer. Wettability anisotropy was obvious in all layers of multi-layered scaffolds. The phenomenon could be explained by the wetting behavior of water droplet on fibrous scaffold. The triple-phase (liquid–air–solid) contact line was influenced by the arrangement of fibers along with the spreading and ingression of water droplet on fibrous topography with air pocket. The water droplet progressed in a periodic stick-slip motion in perpendicular direction and the contact line should overcome the barrier to advance, resulting in a higher WCA value. Less or no barriers to the contact line in aligned direction led to the preferential spreading and ingression of water droplet along the nanofibers and lower WCA value [37].

The mechanical properties of multi-layered electrospun scaffolds in the direction of fiber alignment were determined by using their tensile stress–strain curves which are shown in Fig. 10. The UTS and EM of tri-layered scaffolds were similar to that of tetra-layered scaffolds. However, compared with non-woven fibrous scaffolds with similar composition in another study by our group, the multi-layered scaffolds with aligned-fiber architecture exhibited with much higher EM and UTS but lower EB. The fibers in aligned-fiber and non-woven scaffolds underwent different tensile processes and resulted in the differences in mechanical properties. The stress–strain curves could be divided into two regions—the elastic region and the plastic region. The stress increased sharply in the elastic region with strain less than 4% and peaked at the yielding point, followed by a steady and slow increase until ultimate strength was reached. With the further increase of tensile strain, fibers were stretched and highly aligned along the tensile direction, followed by rupture of individual fibers one after another, resulting in sharp decrease in stress and a large elongation at break [38].

Two plateaus of stress with different durations were evidently noticed in both types of scaffolds before elongation at break. PLGA was tougher and more ductile than PDLLA as flexibility of glycolic acid chain segment in PLGA polymer backbone increased the crystallinity and improved the mechanical properties of PLGA fibers, while the rigidity of lactic acid chain segment and racemization in PDLLA polymer backbone compromised their mechanical properties. A sharp decrease of stress occurred at 30–35% strain which was very likely attributed to fiber breakage of NGF/PDLLA and/or PDLLA fibers with thin fiber diameters as thinner fiber diameter could result in less elongation at break. Therefore, it was speculated that the first plateau of stress was attributed to plastic deformation of both PDLLA fibers with thick fiber diameter and GDNF/PLGA fibers; the second plateau of stress was resulted from the plastic deformation of GDNF/PLGA fibers. The mechanical properties of multi-layered electrospun scaffolds in orthogonal direction of fiber alignment were not investigated in this study. The EM, UTS, and EB of multi-layered scaffolds were similar with or of the same order of magnitude from those of natural soft tissues as well as those of organs consisted of soft connective tissues, suggesting their potential in soft tissue regeneration applications.

Peripheral nerve tissue regeneration is a highly regulated process involving spatial arrangement of different cell types, their dynamic interactions, and neurite differentiation, requiring sustained and multiple delivery of biochemical cues from scaffolds to induce cells and their commitment to regeneration in a spatio-temporal guiding manner [7, 9, 39]. This study demonstrated that the use of multi-layered electrospun scaffolds with multi-layered structures through a combinatorial methodology including sequential electrospinning, HS-ES, DSDP-ES, and emulsion electrospinning, could realize the dual and spatio-temporal delivery of GDNF and NGF with distinct release kinetics. The hydrophobic PDLLA layer with a layer thickness approximately 80 μm composed of PDLLA fibers with thick fiber diameters was utilized as a barrier layer. In tri-layered scaffolds, the release of GDNF and NGF were confined to the two opposite sides of scaffolds. A much faster release of GDNF was achieved as compared to NGF as both diffusion-driven and matrix erosion-driven release contributed to the release of GDNF. Separation efficiency of growth factors was defined as cumulative release amount of growth factors at designed loci over the total cumulative release amount of growth factors. The separation efficiency of GDNF and NGF was both above 93%. In tetra-layered scaffolds, the release of GDNF and NGF were confined to GDNF/PLGA layer side and dual and sequential release of GDNF and NGF was successfully achieved. The separation efficiency of GDNF and NGF were 95 and 89%, respectively. It was speculated that different diffusion distances of GDNF and NGF through fiber matrix and wettability gradient in tetra-layered scaffolds resulted in distinct release kinetics of the two growth factors. The multi-layered fibrous scaffolds constructed and studied in this investigation which could provide dual and spatio-temporal delivery of biochemical cues with distinct release kinetics may also be employed for other tissue regeneration applications [40, 41].

## Conclusions

Multi-layered electrospun scaffolds of large size and with multi-layered structures were successfully produced through a combinatorial methodology including emulsion electrospinning, sequential electrospinning, HS-ES, and DSDP-ES. Aligned fibers were obtained in each layer of multi-layered scaffolds and relatively thick tri-layered and tetra-layered scaffolds with controlled layer thickness were obtained. Dual and spatio-temporal release of GDNF and NGF with distinct release kinetics from multi-layered scaffolds was successfully demonstrated. High separation efficiency by PDLLA fibrous barrier layer for spatial neurotrophic factor delivery from both tri-layered scaffolds and tetra-layered scaffolds was achieved. Taken together, the demonstrated multi-layered scaffolds, capable of providing dual and spatio-temporal delivery of biochemical cues and topographical cues, will benefit scaffold-based tissue regeneration including but not limited to peripheral nerve tissue repair.

## References

[1] Daly W, Yao L, Zeugolis D, Windebank A, Pandit A (2012). A biomaterials approach to peripheral nerve regeneration: bridging the peripheral nerve gap and enhancing functional recovery. J R Soc Interface.

[2] Xie J, MacEwan MR, Liu W, Jesuraj N, Li X, Hunter D, Xia Y (2014). Nerve guidance conduits based on double-layered scaffolds of electrospun nanofibers for repairing the peripheral nervous system. ACS Appl Mater Interfaces.

[3] Beachley V, Wen XJ (2010). Polymer nanofibrous structures: fabrication, biofunctionalization, and cell interactions. Progress Polym Sci.

[4] Georgiou M, Bunting SCJ, Davies HA, Loughlin AJ, Golding JP, Phillips JB (2013). Engineered neural tissue for peripheral nerve repair. Biomaterials.

[5] Koh HS, Yong T, Chan CK, Ramakrishna S (2008). Enhancement of neurite outgrowth using nano-structured scaffolds coupled with laminin. Biomaterials.

[6] Konofaos P, Halen JPV (2013). Nerve repair by means of tubulization: past, present, future. J Reconstr Microsurg.

[7] Catrina S, Gander B, Madduri S (2013). Nerve conduit scaffolds for discrete delivery of two neurotrophic factors. Eur J Pharm Biopharm.

[8] Price TJ, Louria MD, Candelario-Soto D, Dussor GO, Jeske NA, Patwardhan AM, Diogenes A, Trott AA, Hargreaves KM, Flores CM. Treatment of trigeminal ganglion neurons in vitro with NGF, GDNF or BDNF: effects on neuronal survival, neurochemical properties and TRPVI-mediated neuropeptide secretion. BMC Neurosci. 2005;6:4. 10.1186/1471-2202-6-4 PMC548274 15667652

[9] Madduri S, Papaloïzos M, Gander B (2009). Synergistic effect of GDNF and NGF on axonal branching and elongation in vitro. Neurosci Res.

[10] Chew SY, Mi RF, Hoke A, Leong KW (2007). Aligned protein-polymer composite fibers enhance nerve regeneration: a potential tissue-engineering platform. Adv Funct Mater.

[11] Jenkins PM, Laughter MR, Lee DJ, Lee YM, Freed CR, Park D (2015). A nerve guidance conduit with topographical and biochemical cues: potential application using human neural stem cells. Nanoscale Res Lett.

[12] Ruiter GCW, Malessy MJA, Yaszemski MJ, Windebank AJ, Spinner RJ. Designing ideal conduits for peripheral nerve repair. Neurosurg Focus. 2009;26:E5. 10.3171/FOC.2009.26.2.E5 PMC2978041 19435445

[13] Deumens R, Bozkurt A, Meek MF, Marcus MAE, Joosten EAJ, Weis J, Brook GA (2010). Repairing injured peripheral nerves: Bridging the gap. Progress Neurobiol.

[14] Siemionow M, Brzezicki G (2009). Chapter 8: current techniques and concepts in peripheral nerve repair. Int Rev Neurobiol.

[15] Ferrari A, Faraci P, Cecchini M, Beltram F (2010). The effect of alternative neuronal differentiation pathways on PC12 cell adhesion and neurite alignment to nanogratings. Biomaterials.

[16] Tonazzini I, Meucci S, Faraci P, Beltram F, Cecchini M (2013). Neuronal differentiation on anisotropic substrates and the influence of nanotopographical noise on neurite contact guidance. Biomaterials.

[17] Kharaziha M, Nikkhah M, Shin SR, Annabi N, Masoumi N, Gaharwar AK, Camci-Unal G, Khademhosseini A (2013). PGS:Gelatin nanofibrous scaffolds with tunable mechanical and structural properties for engineering cardiac tissues. Biomaterials.

[18] Nista SVG, Bettini J, Mei LHI (2015). Coaxial nanofibers of chitosan–alginate–PEO polycomplex obtained by electrospinning. Carbohydr Polym.

[19] Park SY, Ki CS, Park YH, Lee KG, Kang SW, Kweon HY, Kim HJ (2015). Functional recovery guided by an electrospun silk fibroin conduit after sciatic nerve injury in rats. J Tissue Eng Regen Med.

[20] Xue J, He M, Liu H, Niu Y, Crawford A, Coates PD, Chen D, Shi R, Zhang L (2014). Drug loaded homogeneous electrospun PCL/gelatin hybrid nanofiber structures for anti-infective tissue regeneration membranes. Biomaterials.

[21] Zhang Y, Lei Y, Chang J, Li L, He B, Gu ZW (2012). Guidance of myoblast migration on aligned electrospun PLGA nanofibrous meshes. Mater Lett.

[22] Xu H, Cui WG, Chang J (2013). Fabrication of patterned PDLLA/PCL composite scaffold by electrospinning. J Appl Polym Sci.

[23] Llorens E, Ibañez H, del Valle LJ, Puiggalí J (2015). Biocompatibility and drug release behavior of scaffolds prepared by coaxial electrospinning of poly(butylene succinate) and polyethylene glycol. Mater Sci Eng: C.

[24] Puhl S, Ilko D, Li LH, Holzgrabe U, Meinel L, Germershaus O (2014). Protein release from electrospun nonwovens: Improving the release characteristics through rational combination of polyester blend matrices with polidocanol. Int J Pharm.

[25] Wang XY, Yuan YH, Huang XC, Yue TL (2015). Controlled release of protein from core-shell nanofibers prepared by emulsion electrospinning based on green chemical. J Appl Polymer Sci.

[26] Yau WWY, Long HY, Gauthier NC, Chan JKY, Chew SY (2015). The effects of nanofiber diameter and orientation on siRNA uptake and gene silencing. Biomaterials.

[27] Liu CY, Wang C, Zhao QL, Li XH, Xu FY, Yao XM, Wang Liu M (2018). Incorporation and release of dual growth factors for nerve tissue engineering using nanofibrous bicomponent scaffolds. Biomed Mater.

[28] Chew SY, Mi R, Hoke A, Leong KW (2008). The effect of the alignment of electrospun fibrous scaffolds on Schwann cell maturation. Biomaterials.

[29] Wang CY, Zhang KH, Fan CY, Mo XM, Ruan HJ, Li FF (2011). Aligned natural-synthetic polyblend nanofibers for peripheral nerve regeneration. Acta Biomater.

[30] Li A, Hokugo A, Yalom A, Berns EJ, Stephanopoulos N, McClendon MT, Segovia LA, Spigelman I, Stupp SI, Jarrahy R (2014). A bioengineered peripheral nerve construct using aligned peptide amphiphile nanofibers. Biomaterials.

[31] Matthias MLA, Christian G, Helga B, Heinrich S (2012). Electrospinning of aligned fibers with adjustable orientation using auxiliary electrodes. Sci Technol Adv Mater.

[32] Yang DZ, Zhang JF, Zhang J, Nie J (2008). Aligned electrospun nanofibers induced by magnetic field. J Appl Polym Sci.

[33] Liu YQ, Zhang XP, Xia YN, Yang H (2010). Magnetic-field-assisted electrospinning of aligned straight and wavy polymeric nanofibers. Adv Mater.

[34] Theron A, Zussman E, Yarin AL (2001). Electrostatic field-assisted alignment of electrospun nanofibres. Nanotechnology.

[35] Tong HW, Wang M (2011). An investigation into the influence of electrospinning parameters on the diameter and alignment of Poly(hydroxybutyrate-co-hydroxyvalerate) fibers. J Appl Polym Sci.

[36] Hakamada Y, Ohgushi N, Fujimura-Kondo N, Matsuda T (2012). Electrospun Poly(gamma-Benzyl-L-Glutamate) and its alkali-treated meshes: their water wettability and cell-adhesion potential. J Biomater Sci-Polym Ed.

[37] Wu H, Zhang R, Sun Y, Lin DD, Sun ZQ, Pan W, Downs P (2008). Biomimetic nanofiber patterns with controlled wettability. Soft Matter.

[38] Shi QF, Zhou CJ, Yue YY, Guo WH, Wu YQ, Wu QL (2012). Mechanical properties and in vitro degradation of electrospun bio-nanocomposite mats from PLA and cellulose nanocrystals. Carbohydr Polym.

[39] Bonani W, Motta A, Migliaresi C, Tan W (2012). Biomolecule gradient in micropatterned nanofibrous scaffold for spatiotemporal release. Langmuir.

[40] Masoumi N, Annabi N, Assmann A, Larson BL, Hjortnaes J, Alemdar N, Kharaziha M, Manning KB, Mayer JE, Khademhosseini A (2014). Tri-layered elastomeric scaffolds for engineering heart valve leaflets. Biomaterials.

[41] Zhang H, Jia XL, Han FX, Zhao J, Zhao YH, Fan YB, Yuan XY (2013). Dual-delivery of VEGF and PDGF by double-layered electrospun membranes for blood vessel regeneration. Biomaterials.

